# Supplementary material for: Evidence that Entomophthora muscae controls the timing of host death via its own circadian clock
Source: bioRxiv. 2025 Jun 18:2025.06.18.660419. Preprint. [Version 1] doi: 10.1101/2025.06.18.660419 (PMC12262267; doi:10.1101/2025.06.18.660419)
Supplement: Supplement 2 [file NIHPP2025.06.18.660419v1-supplement-2.pdf]

bioRxiv preprint doi: https://doi.org/10.1101/2025.06.18.660419; this version posted June 18, 2025. The copyright holder for this preprint (which was not certified by peer review) is the author/funder, who has granted bioRxiv a license to display the preprint in perpetuity. It is made available under aCC-BY 4.0 International license.

Supplementary Figures and Tables

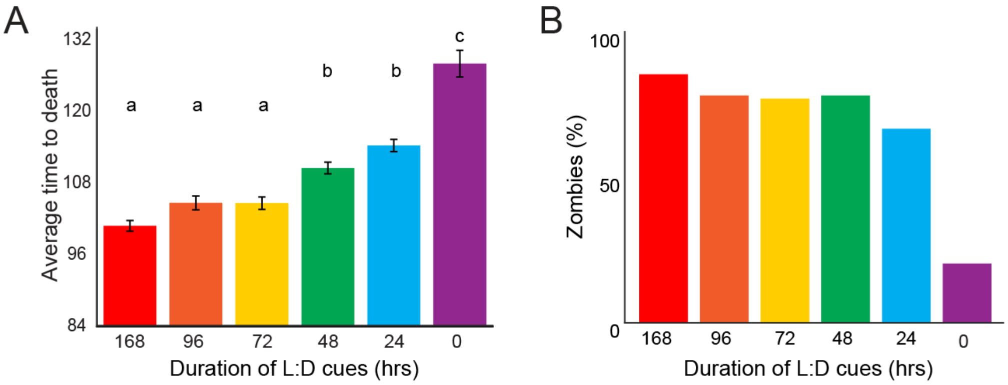

Figure 1-S1. Observations in WT flies exposed to *E. muscae*. A) Mean time until death for WT (CantonS) flies exposed to *E. muscae* over a range of lighting conditions. Error bars are SEM. Letters above bars indicate significance groups ($p < 0.05$) as determined by comparisons (two-tailed t-test) for each experiment pair. B) Percentage of *E. muscae*-exposed flies that became zombies in **Figure 1A**.

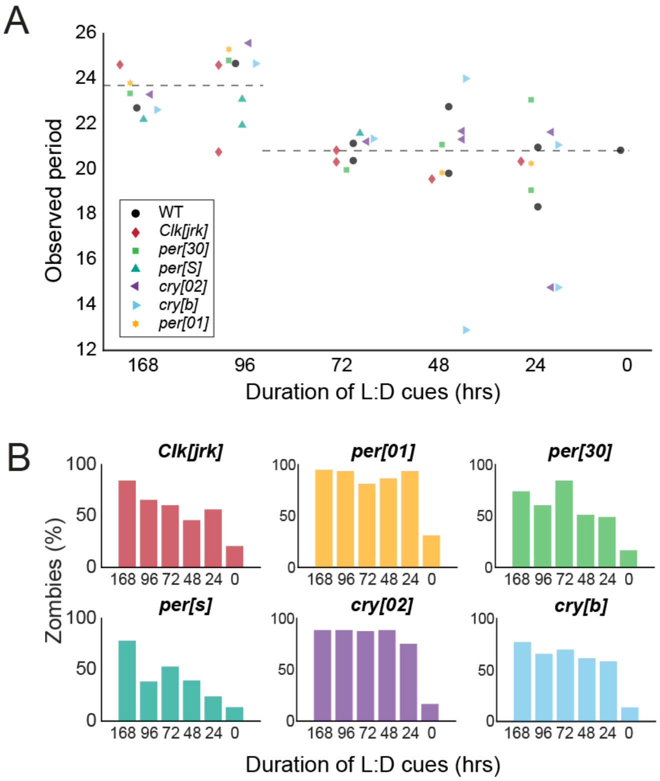

Figure 3-S1. Observations in circadian mutants exposed to *E. muscae*. A) Observed death period lengths (in hours) for flies in Fig. 3B-G. For every day in which at least five flies were observed to die, the mean time of death was calculated; the difference between mean times of death across consecutive days is plotted. Dashed line at left reflects mean observed period for experiments where L:D cues were provided for experiments in which flies had at least one death day where L:D cues were provided (168 and 96 hours after *E. muscae* exposure; 23.8 hours); at right, mean for all experiments where flies were housed in D:D across all days of death (20.4 hours). Two-tailed t-test shows significant difference between L:D and D:D periods (p = 7.3e-05 excluding Cs WF periods, 5.5e-06 including Cs WF periods). B) Percentage of exposed flies that died and sporulated following *E. muscae* infection for flies in Fig. 3B-G.

bioRxiv preprint doi: https://doi.org/10.1101/2025.06.18.660419; this version posted June 18, 2025. The copyright holder for this preprint (which was not certified by peer review) is the author/funder, who has granted bioRxiv a license to display the preprint in perpetuity. It is made available under aCC-BY 4.0 International license.

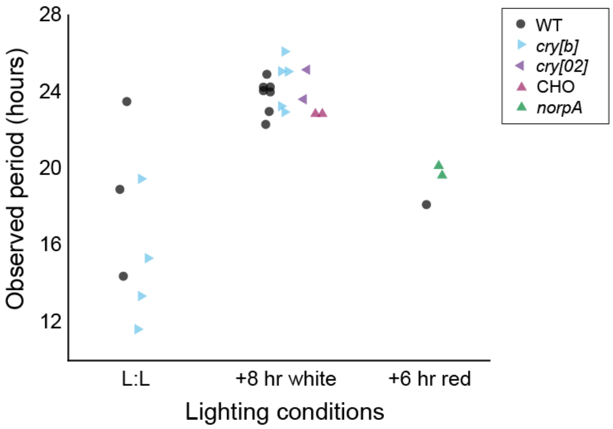

Figure 4-S1. Observed death period lengths (in hours) for experiments in Figure 4. For every day in which at least five flies were observed to die, the mean time of death was calculated and plotted above. Data from all replicates (not just representative experiments shown in Figure 4) were used.

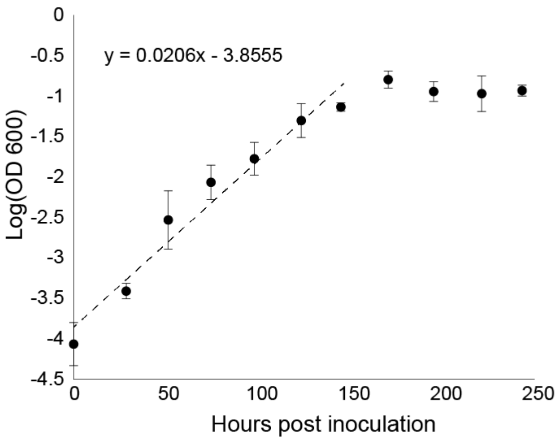

Figure 5-S1. *In vitro E. muscae* growth curve at room temperature. *E. muscae* was at room temperature; OD600 was measured every ~24 hours over 11 days (see Methods). Each dot represents the mean value of the triplicate cultures measured in triplicate; error bars show standard deviation between these values. Equation for line of best fit in linear region shown above; doubling over this time occurs every 30 hours on average.

bioRxiv preprint doi: https://doi.org/10.1101/2025.06.18.660419; this version posted June 18, 2025. The copyright holder for this preprint (which was not certified by peer review) is the author/funder, who has granted bioRxiv a license to display the preprint in perpetuity. It is made available under aCC-BY 4.0 International license.

Table S1. Functional groupings and phasing of cycling genes.

Group	Gene	Putative ID	Features^	LD phase*	DD phase*
metabolism	*DSO57_1002569*	DESI1		8.8	9.2
signaling	*DSO57_1002592*	KinE-superfamily TMAO_torS superfamily		12.5	11.1
transport	*DSO57_1002842*	Vnx1p		11.8	9.5
transport	*DSO57_1003020*	KIF11_1		12.9	11.8
DNA binding	*DSO57_1003339*	CCHC-type zinc finger protein		13.2	11.9
RNA processing	*DSO57_1003661*	Tho3		12.1	11.5
chaperones	*DSO57_1003835*	DnaJ		11.6	10.7
unknown	*DSO57_1004864*	Unknown	signal peptide	21.1	1.8
chaperones	*DSO57_1007651*	HSP-20 family		11.9	11.0
chaperones	*DSO57_1007654*	HSP-20 family		11.9	10.9
transport	*DSO57_1007682*	NTF2_2		11.5	10.7
metabolism	*DSO57_1007850*	Peptidase S1	signal peptide	11.7	11.9
RNA processing	*DSO57_1008787*	Ribonuclease Z		12.1	11.9
unknown	*DSO57_1009136*	Unknown	signal peptide	1.1	1.2
metabolism	*DSO57_1009172*	AMD1_2		11.5	11.7
DNA binding	*DSO57_1009395*	FAR1 domain		9.6	8.2
DNA binding	*DSO57_1011895*	FAR1 domain		1.0	6.6
unknown	*DSO57_1012176*	Unknown	signal peptide	9.2	12.1
unknown	*DSO57_1013256*	Unknown	GPCR-like	11.9	15.9
unknown	*DSO57_1014401*	Unknown	transmembrane	9.9	14.4
chaperones	*DSO57_1014412*	HSP-20 family		11.9	11.0
metabolism	*DSO57_1014762*	NADP-ME		11.3	11.4
RNA processing	*DSO57_1016884*	LAS1_1		12.0	11.8
unknown	*DSO57_1016932*	Unknown	signal peptide	0.0	0.0
transport	*DSO57_1017186*	MYO2_4		11.3	11.8
DNA binding	*DSO57_1018521*	Gal4 superfamily		2.1	5.4
DNA binding	*DSO57_1019068*	CCHC-type zinc finger protein		13.0	12.0
unknown	*DSO57_1020463*	Unknown	signal peptide	8.1	11.2

bioRxiv preprint doi: https://doi.org/10.1101/2025.06.18.660419; this version posted June 18, 2025. The copyright holder for this preprint (which was not certified by peer review) is the author/funder, who has granted bioRxiv a license to display the preprint in perpetuity. It is made available under aCC-BY 4.0 International license.

metabolism	*DSO57_1021015*	GCD		9.7	9.5
transport	*DSO57_1024668*	DRS2_2		11.5	11.1
chaperones	*DSO57_1025376*	STI1_4		11.6	10.6
unknown	*DSO57_1026842*	Unknown	transmembrane	13.3	13.4
transport	*DSO57_1026944*	MFS transporter		11.7	13.5
chaperones	*DSO57_1028158*	HSP-20 family		11.9	11.0
chaperones	*DSO57_1028384*	SWA2_1		10.4	11.7
DNA binding	*DSO57_1029495*	FAR1 domain		0.0	6.8
metabolism	*DSO57_1029821*	GUP1_1		12.9	11.6
metabolism	*DSO57_1030034*	ICL1_2		20.1	20.0
chaperones	*DSO57_1030297*	HSP-20 family		11.8	11.0
unknown	*DSO57_1030539*	Unknown	GPCR-like	11.9	15.8
unknown	*DSO57_1030541*	Unknown	GPCR-like	11.8	16.9
chaperones	*DSO57_1030771*	calnexin		12.1	12.0
signaling	*DSO57_1031378*	dSlo	GPCR	8.3	10.4
chaperones	*DSO57_1033354*	LONRF		9.2	9.6
unknown	*DSO57_1033975*	Unknown	GPCR-like	10.0	13.9
metabolism	*DSO57_1034273*	AKR7A family		10.2	11.8
DNA binding	*DSO57_1034661*	FAR1 domain		10.1	9.6
chaperones	*DSO57_1034776*	HSP-20 family		11.9	11.0
DNA binding	*DSO57_1035137*	Myb protein family		11.7	11.8
chaperones	*DSO57_1036078*	HSP-20 family		11.9	11.0
chaperones	*DSO57_1036198*	HSP-20 family		11.8	10.9
chaperones	*DSO57_1036199*	HSPA-1		11.7	10.9
signaling	*DSO57_1037547*	WC-1 homology	WC-1 homology	8.9	10.8
transport	*DSO57_1037577*	MFS transporter		11.4	13.4
DNA binding	*DSO57_1038705*	Gal4 superfamily		8.2	9.3
metabolism	*DSO57_1038916*	AOX2_2		13.0	11.8
unknown	*DSO57_1039705*	Unknown	transmembrane	11.7	11.8

^Full details available in **Supplementary File S2**.
*As calculated by meta2d_phase.

bioRxiv preprint doi: https://doi.org/10.1101/2025.06.18.660419; this version posted June 18, 2025. The copyright holder for this preprint (which was not certified by peer review) is the author/funder, who has granted bioRxiv a license to display the preprint in perpetuity. It is made available under aCC-BY 4.0 International license.

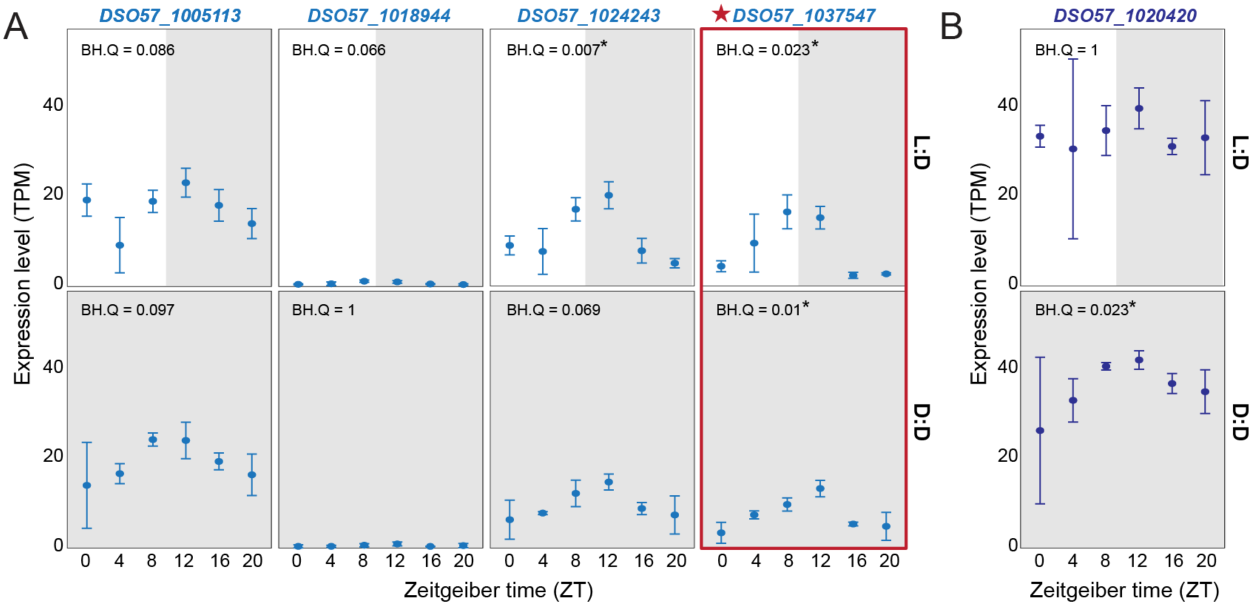

Figure 5-S2. Expression patterns of *N. crassa* circadian homologs. Expression (in transcripts per million [TPM]) for A) each *white collar-1* homolog and B) the one *white collar-2* homolog across samples. Benjamini Hochberg Q (BH.Q) value is noted for each gene. BH.Q values below 0.05 are marked with an asterisk. To be considered a cycler (for Figure 5), BH.Q needed to be less than 0.05 across both L:D and D:D conditions. The one gene whose transcription pattern meets these criteria (*DSO57_1037547*) is outlined in red.

Supplementary Files

Supplemental File S1. MetaCycle results for *in vitro E. muscae* time course.

Supplemental File S2. Annotations for predicted cycling genes (FDR = 5%).
